# Data correction and verification of thermal simulation experiments under the influence of bulging belly

**DOI:** 10.1038/s41598-023-43129-3

**Published:** 2023-09-29

**Authors:** Bing Zheng, Dong Xu, Zhipeng Zou, Yiqun Wang, Longxin Guo, Hongyang Zhao, Dongying Ju

**Affiliations:** 1 School of Materials and Metallurgy, University of Science and Technology Liaoning, Anshan, 114051 China; 2 Technology Innovation Center for High Quality Cold Heading Steel of Hebei Province, Hebei University of Engineering, Handan, 056038 China; 3 Whole Processes of High Quality Fastener Application Technology Research Center of Universities in Hebei Province, Hebei University of Engineering, Handan, 056038 China; 4 Technology Center, Henan Zhongyuan Special Steel Equipment Manufacturing Co. Ltd, Jiyuan, 459008 China; 5 Engineering Research Center for High Toughness Wind Tower Steel of Hebei Province, Hebei Puyang Iron and Steel Co., Ltd., Handan, 056305 China

**Keywords:** Mechanical engineering, Theory and computation

## Abstract

During the thermal simulation compression test, the formation of an obvious bulge in the specimen leads to a certain deviation between the calculated and actual values of the true stress. The finite element method was used to simulate the single-pass compression of specimens of 34CrNi3MoV steel and obtain the actual nonuniform deformation of the bulging belly during the compression process, and the results were applied to correct experimental flow curves. The results showed that the deformation conditions had a significant influence on the nonuniformity of the specimen deformation during the compression process, and all the modified flow curves were lower than the original ones. The size of the bulge and the metal flow line in the finite element simulation were consistent with the test results. The load value obtained by using the modified flow curve was similar to the load value measured in the test, which indicated that the modified flow curve was very close to the real flow force curve of the material. The method used to modify the flow force curve is simple and practical.

## Introduction

Thermal experiments are a scientific approach and important engineering technique to investigate the thermal deformation of metal materials. Experiments with small specimens can reveal trends in the changes of the mechanical properties of materials during the process of thermoplastic deformation. Finite element simulations of the deformation of materials during thermal experiments are important for research, and the results have important academic significance and engineering value.

The flow stress obtained from the thermal simulation experiment can not only reflect the performance of the material during processing in a high temperature state but also manifest the change in the microstructure of the material during thermal deformation^1,2^. At present, many scholars use the control variable method to obtain the flow curve and apply the flow curve as the basis for further research on the thermal deformation behavior of the material and the mechanism of tissue evolution^3–5^. However, during the thermal simulation experiment, the true stress value of the flow curve is calculated under the condition of uniform deformation of the material, which is inconsistent with the actual condition of the material under compression.

Under actual compression, the material undergoes friction and a temperature gradient. As a result, the compression process causes the formation of an obvious deformation or “belly”. That is, the material is nonuniform, which directly affects thermal experiments and results for the true stress. This is the root cause of a certain deviation in the value of the true stress^6,7^, and it is likely that this deviation directly affects the further study of the material. Thus, it is very necessary to study methods to obtain true flow curves of materials.

To address this situation, many scholars conducted systematic studies on the correction of flow curves by considering the effects of friction and temperature rise. Wang^8^ corrected the flow curves of Ti60 alloy, step by step, by using a correction formula and concluded that the equivalent friction correction coefficient had a linear relationship with the strain, and the difference between the flow stress before and after the friction correction increased with increasing strain. Shang^9^ concluded that there are many unavoidable systematic errors when the material is subjected to compression testing. Thus, it is necessary to apply a friction correction and a temperature correction to the flow stress curves derived from the test. The corresponding constitutive model and thermal processing diagram are established by using the corrected flow curve. Hu^10^ claimed that the adiabatic heating of the specimen and the interfacial friction between the anvils had a great influence on the mechanical properties of the material in compression processing and that the failure to take these two factors into consideration may lead to an uncertain error between the test and actual values. Zhu^4^ noted that the friction between the sample and the mold during the compression process must be considered and that is was difficult to completely eliminate the friction even by the use of lubricant. Therefore, after correcting the flow curves obtained from the tests and based on the dynamic material model, the thermal processing diagram of 2050Al-Li2 was established.

In current research on correction of flow curves, the main focus is on the use of correction formulas to eliminate the influence of the effects of friction and temperature rise on the flow stress. However, most of the correction formulas are empirical formulas with different correction parameters for different materials, which have certain limitations. Additionally, there is no method available to verify the accuracy of the corrections of the flow curves. Thus, it is necessary to propose a universal and verifiable correction method for flow curves.

34CrNi3MoV steel has good comprehensive mechanical properties and is one of the important steel materials used commonly in applications for strategic national defense; it has very high performance requirements but is very difficult to forge. Therefore, before thermal processing, numerical simulation is usually used to evaluate and optimize the process^11–13^. The rheology curve is one of the most basic types of data used in research on thermal processing, and its accuracy largely determines the reliability of the simulation results^14–16^. Therefore, in this study, taking 34CrNi3MoV steel as the research object, the flow curve data obtained from single-pass thermal compression experiments were input into the material library of the finite element simulation program, and the material compression process was simulated. The actual area of the deformation of the bulge belly during the compression process was obtained by point tracking. Finally, the ratio of the load in the test to the area of the bulge belly in the simulation was used to repeat the calculation to obtain more accurate flow stress data, correct the flow curve and obtain the value of the true stress that converged to the real situation. The ultimate goal was to provid more accurate basic data for the numerical simulation of 34CrNi3MoV steel.

## Experiment

**Figure 1:**
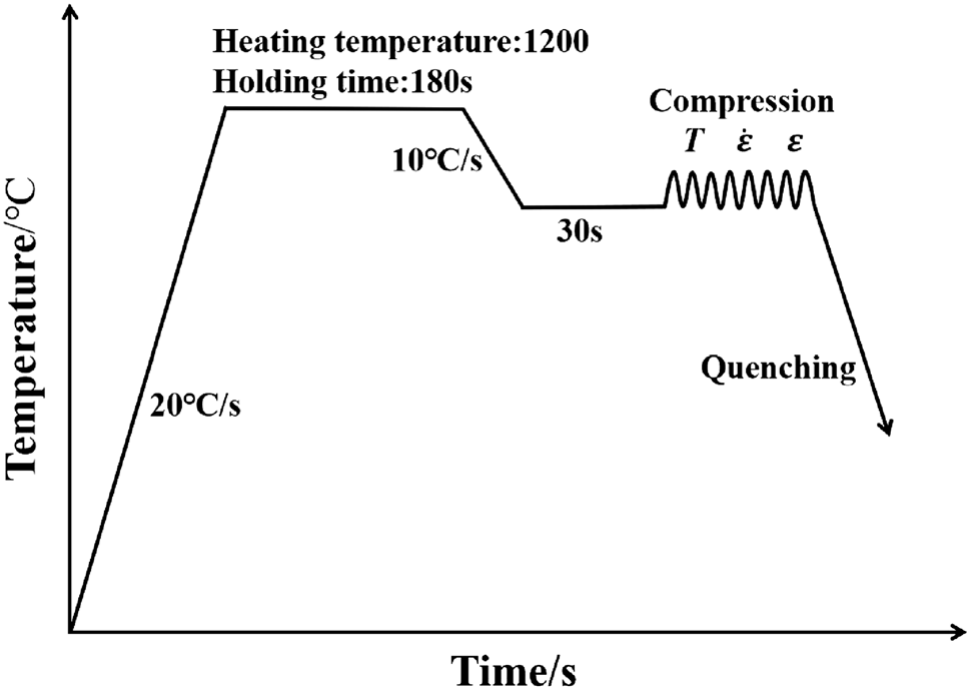
Process flow chart of the isothermal compression test of 34CrNi3MoV steel.

The test sample was made of 34CrNi3MoV steel after industrial hot rolling. The size of the compressed sample was Φ8 mm × 12 mm. The chemical composition is shown in Table 1. The process diagram of the single-pass compression process is shown in Fig. 1. Under the action of resistance heating, the sample was heated at 20 °C/s, then heated at 1200 °C for 180 s to ensure complete austenitization of the sample, cooled at 10 °C/s to the deformation parameter temperature and kept for 30 s to ensure a uniform temperature distribution in the sample. Finally, the compression test was completed. The deformation parameters of the compression process were: temperature set at 800, 900, 1000, 1100 and 1200 °C, strain rate 0.01, 0.1, 1 and 10 s^−1^, and maximum deformation of 50% (true strain 0.6931). The compressed sample was quenched with water, and the grain morphology was observed by metallographic microscopy (Olympus).

**Table 1:** Chemical composition of 34CrNi3MoV steel (%, mass fraction).

C	Si	Mn	Cr	Ni	Mo	V	Cu	P	S	Fe
0.28–0.35	0.15–0.4	0.5–0.8	1.2–1.5	3.0–3.3	0.35–0.45	0.1–0.2	≤ 0.02	≤ 0.015	≤ 0.001	Bal

## Result and analysis

### Flow curve

In the isothermal compression experiment, to obtain the true stress-true strain curve, a data system was used to collect the values of the displacement sensor and pressure sensor during the experiment and analyze the collected data to obtain the values of the true strain and true stress. The calculation formula is shown in Eqs. (1) and (2):

1 $$\varepsilon = \ln \left( {\frac{{l_{0} + \Delta l}}{{l_{0} }}} \right)$$

2 $$\sigma = \frac{F}{S} = \frac{F}{{\frac{{\frac{{\pi \left( {d_{0} } \right)^{2} }}{4}l_{0} }}{{l_{0} + \Delta l}}}}$$

where *l*_0_ is the initial length of the sample, mm; Δ*l* is the displacement during specimen compression, mm; *ε* is the dependent variable; *S* is the cross-sectional area of the sample, mm^2^; *F* is the experimental load, kgf; *d*_0_ is the initial diameter of the sample, mm; and *σ* is the stress, MPa.

**Figure 2:**
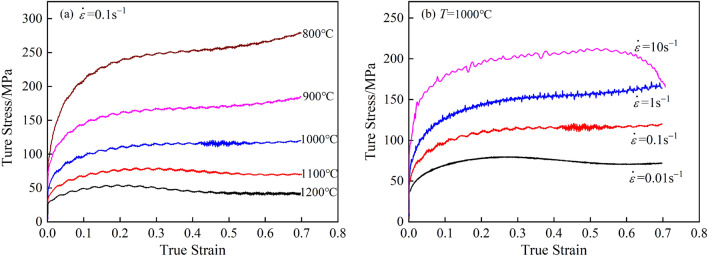
True stress‒strain curves of 34CrNi3MoV steel under different deformation conditions. (**a**) 0.1 s^−1^; (**b**) 1000 °C

**Figure 3:**
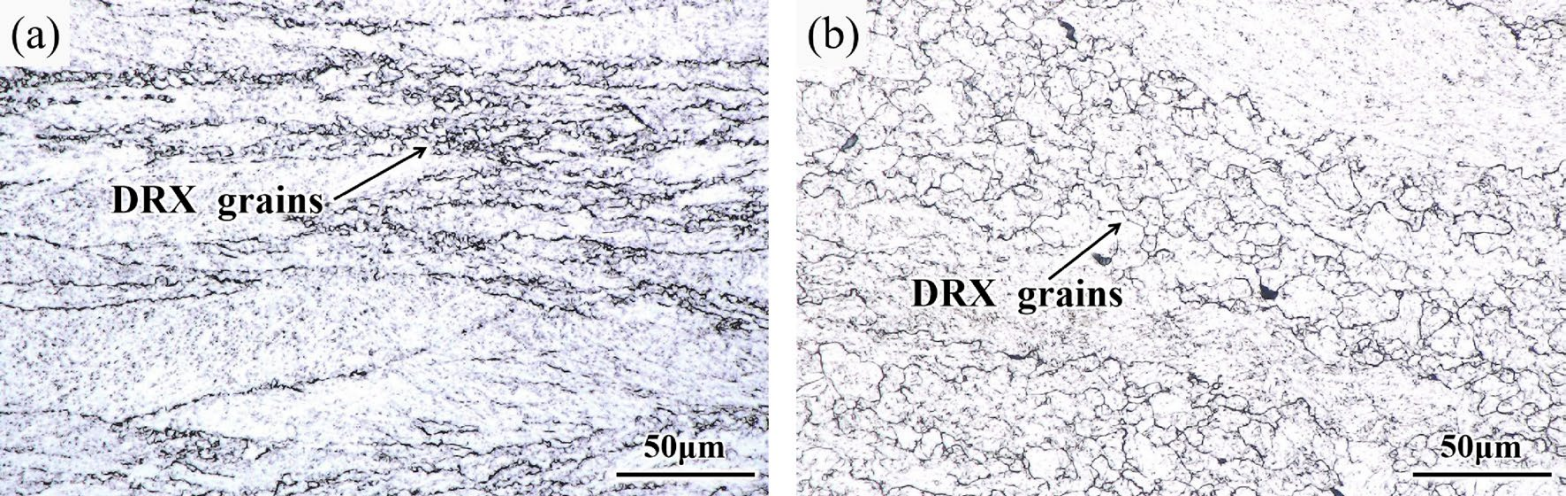
Microstructure of 34CrNi3MoV steel. (**a**) 900 °C, 0.1 s^−1^; (**b**) 1000 °C, 10 s^−1^.

Figure 2 shows the true stress‒strain curves of 34CrNi3MoV steel under different deformation conditions calculated by Eqs. (1) and (2) after the experiment. Figure 2a shows the flow curve at each deformation temperature at a strain rate of 0.01 s^−1^. The peak stress did not appear at 900 °C and 1000 °C. Figure 2b shows the flow curve at various strain rates at a deformation temperature of 1000 °C. When the strain rate was 10 s^−1^, early unloading occurred due to the fast compression process and difficult operation. At the same time, there was no peak stress when the strain rate was 1 s^−1^ and 10 s^−1^. However, the metallographic observations under the above compression conditions found that dynamic recrystallization (DRX) occurred in all of the samples. As shown in Fig. 3, the deformation conditions were 0.1 s^−1^ at 900 °C and 10 s^−1^ at 1000 °C, and DRX occurred in all of them.

This was due to the friction between the indenter and the specimen end face in the actual compression experiment, as well as the presence of a certain temperature gradient inside the specimen during the compression process. Thus, the deformation of the specimen during the compression process was uneven; that is, there was a certain bulge in the specimen during the compression process. Therefore, when using Eq. (2), there was some error in the calculation method of the cross-sectional area *S*, and the calculated value of the true stress deviated from the actual value.

If the flow line curve data calculated by Eq. (2) were directly used, then the conclusion of deciding whether DRX occurred by direct use of the peak stress would be wrong, and the establishment of the later constitutive model and DRX-related model would also deviate from the true value, resulting in greater errors. Therefore, it was necessary to eliminate the influence of error caused by the bulge phenomenon.

In this study, finite element simulation software were used to simulate the experimental process, the actual area of the middle cross section of the specimen in the compression process was obtained by point tracing, and then the flow curve was corrected.

### Flow curve correction process

#### Establishment of the finite element simulation model

**Figure 4:**
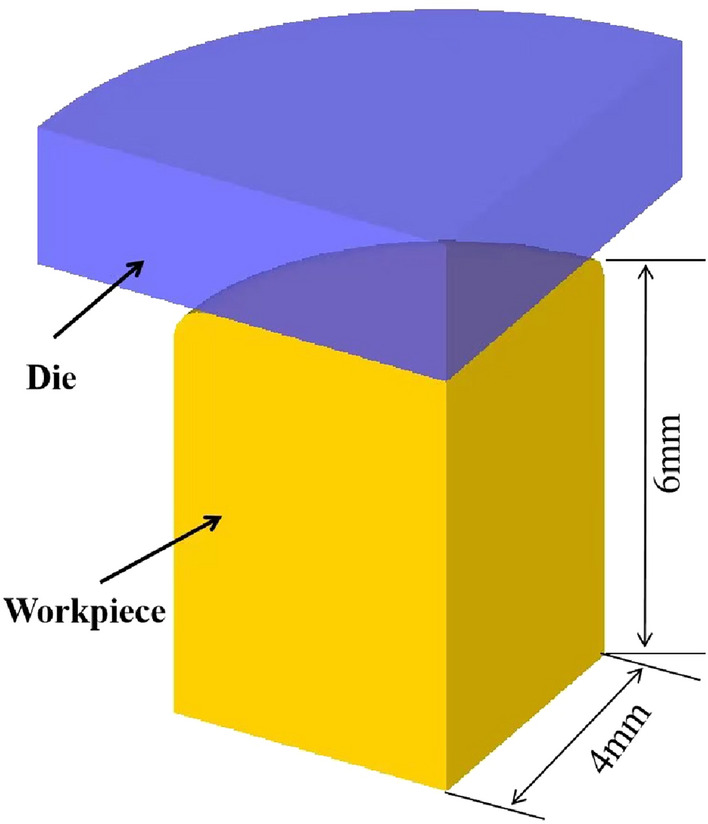
Numerical simulation of the 3D model.

A 3D model was established with the mapping function of the simulation software. As shown in Fig. 4, 34CrNi3MoV steel was used as the workpiece material, the stress‒strain curve of the material was experimental data, and other material constants were calculated by JMatPro software. To reduce the simulation time, 1/8 of the cylindrical shape was calculated.

In the simulation process, the simulation step size was set to 0.1 mm, the workpiece had a plastic body, the initial temperature was the same as the deformation temperature of the test design, and the heat transfer coefficient between the workpiece and the environment was 0.01 (N/sec/mm/°C). The mold was a rigid body, and the initial temperature was 20 °C. Since the size of the drum belly was related to the internal temperature of the specimen and the interfacial friction, the friction coefficient f and the transfer coefficient n between the workpiece and the mold were initially explored by using the control variable method before simulation of the test process, the friction coefficient between the workpiece and the mold was ultimately set to 0.3, and the transfer coefficient was 1 (N/sec/mm/°C). The simulation compression process used speed to control the movement of the die. Since the workpiece was set up with upper and lower symmetry planes, the conversion formula of strain rate and speed is shown in Eq. (3), which is the functional relationship between speed and stroke X-axis.

3 $$v = \frac{1}{2}\dot{\varepsilon }\left( {h - s} \right)$$

where $\dot{\varepsilon }$ is the strain rate, s^−1^; *h* is the height of the sample before compression, mm; and *s* is the compression stroke X-axis of the upper die under each deformation parameter, mm.

#### Calculation of the area of the bulge

**Figure 5:**
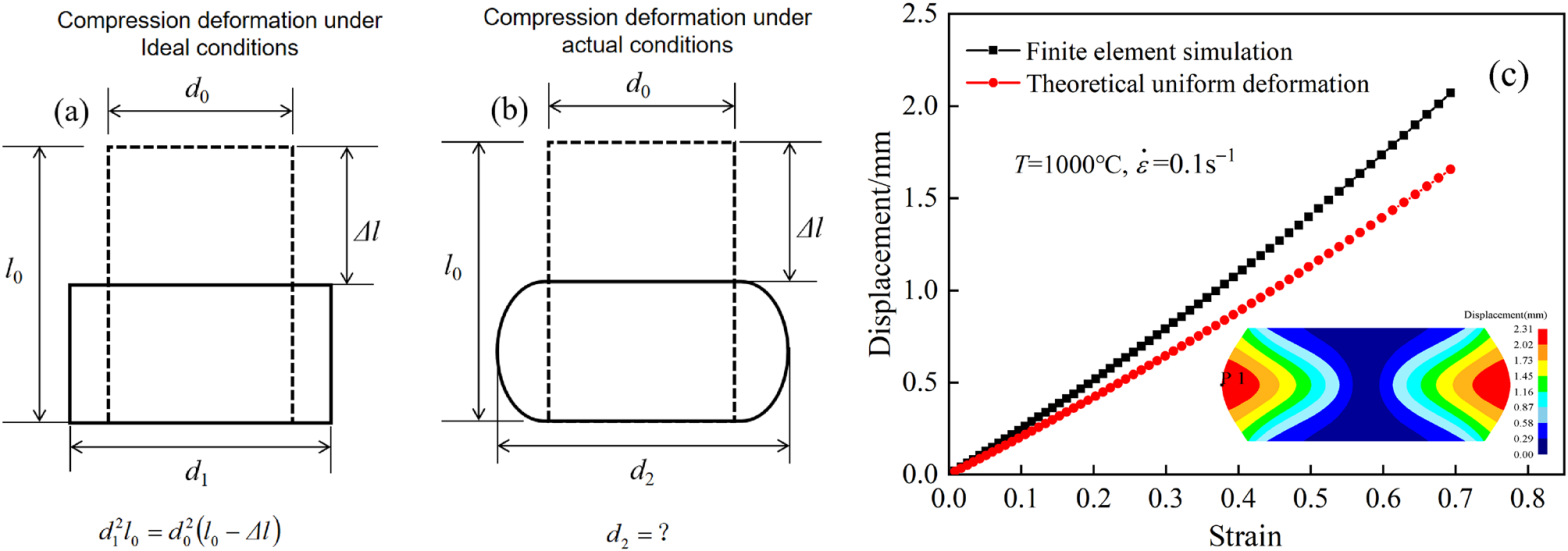
Displacement distribution of nodes. (**a**) Theoretical uniform conditions; (**b**) actual conditions; (**c**) simulated deformation (*T* = 1000 °C, $\dot{\varepsilon }$ = 0.1 s^−1^).

Through the finite element simulation, the distribution of the horizontal displacement of each node of the sample during compression was obtained, as shown in Fig. 5. Figure 5a shows the deformation under the theoretical homogeneous conditions, where it was easy to calculate the deformed diameter *d*_1_, and the thermal experiments are used to directly calculate the stress value of the flow curve. Figure 5b shows the deformation under the actual compression conditions, where *d*_2_ is the actual diameter of the drum belly. It was difficult to confirm the diameter *d*_2_, but it was the actual diameter of the actual compression process. Therefore, it was necessary to simulate the actual compression process through the finite element software. Figure 5c shows the results of the simulation of the deformation under actual deformation conditions where which point tracking was used to obtain the size of the actual diameter during the whole process.

According to the distribution cloud map at the lower right corner of Fig. 5c, the horizontal displacements of the upper and lower ends of the sample after compression were almost zero because there was large friction between the sample and the indenter, and it was difficult for the upper and lower ends of the sample to deform in the horizontal direction during the compression process. However, the central part of the sample was subjected to external forces along the vertical direction, and the grains in this region were flattened. When the deformation increases, the grains flowed to both sides, causing the compressed sample to form a belly bulge, and the displacement value at the P1 point of the central cross section reached the maximum.

To calculate the area of the central cross section of the compressed sample under different amounts of compression, the horizontal displacement value of point P1 under different compression amounts was obtained by using point tracing. The curve in Fig. 5c shows the results of the data collected at the deformation condition of 1000 °C and 0.1 s^−1^. The displacement of the node at the center cross section increased with increasing compression. Meanwhile, the simulation results showed that the displacement of tracking point P1 under actual compression was significantly larger than the theoretical displacement during homogeneous deformation, and the difference between the two gradually increased with increasing compression. Under the deformation conditions, the finite element simulation calculated that the maximum displacement of point P1 was 2.07 after completing the compression test.

**Figure 6:**
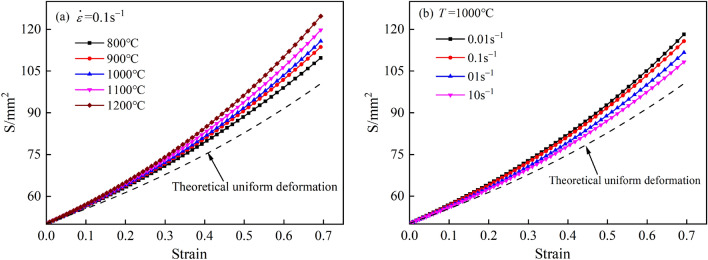
Relationship between intermediate cross-sectional area and compressive strain under different deformation conditions. (**a**) 0.1 s^−1^; (**b**) 1000 °C.

According to the above results for the point tracking displacement, the relationship diagram was calculated for the change in the cross-sectional area S at the bulge belly with increasing compression under different deformation conditions. However, due to the large fluctuations in the load data at the later stage of the hot compression experiment, the deformation in the actual process was less than 50% (the dependent variable was 0.6931). Therefore, in this study, only statistical calculations were conducted for data with variables in the range of X-axis. The results are shown in Fig. 6, where the dashed line is the theoretical area value under uniform deformation. The area of the belly drum during the actual compression process was larger than the theoretical area under uniform deformation, and the difference between the two gradually increased with increasing strain, indicating that there was an obvious inhomogeneity in the deformation of the specimen in the actual compression process, and the inhomogeneity was more obvious with increasing compression. As shown in Fig. 6a, the cross-sectional area at the bulge tended to increase with increasing deformation temperature. This was because under the same compression rate, the higher the temperature was, the greater the internal plasticity of the metal, and the stronger the material fluidity was during compression, the more obvious the bulge became, resulting in a larger cross-sectional area at the center. Figure 6b shows that at the same deformation temperature, the cross-sectional area at the drum belly increased with decreasing strain rate. This was because the smaller the strain rate was during compression, the more time it took for the material to complete the same amount of compression, and there was sufficient time for the metal to flow. Thus, the internal temperature gradient of the sample was larger, and the cross-sectional area at the bulge belly was larger under the double action^17^.

#### Comparison of flow curves after finite element modification

**Figure 7:**
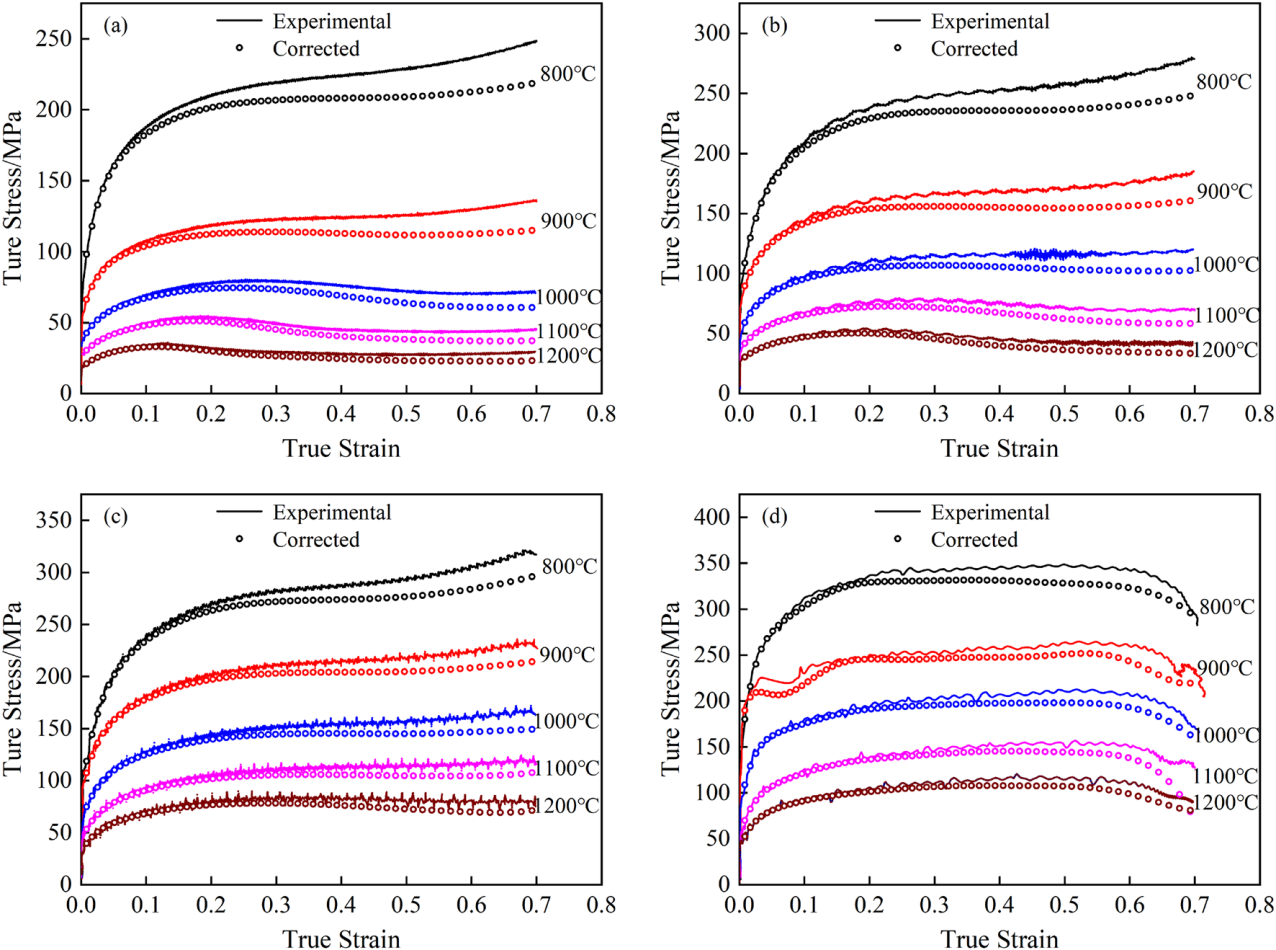
Comparison between the original flow curves and the modified finite element flow curves. (**a**) 0.01 s^−1^; (**b**) 0.1 s^−1^; (**c**) 1 s^−1^; (**d**) 10 s^−1^.

Since the load in the compression test was calculated directly from the pressure transducer for the pressure value in the vertical direction, the ratio of the load obtained from the test to the central cross-sectional area was used to calculate the true stress, which enabled the correction of the flow curve. Figure 7 shows the comparison between the original flow curve and the modified flow curve of the finite element. As shown in Fig. 7, all the flow curves modified by the finite element method were lower than the original flow curves. At the initial stage of deformation, the difference between the two was small, but with increasing deformation, the difference between the two gradually increased.

To more clearly describe the error before and after correction, the mean relative error (*R*_AV_) of the flow curve before and after correction was calculated and statistically analyzed, and the results are shown in Table 2. When the deformation temperature increased, the strain rate decreased, the compression true strain increased, the average error gradually increased, and all these errors were caused by the obvious bulge formed during the compression process. When the deformation temperature rose from 800 to 1200 °C, the *R*_AV_ value rose from 4.54 to 7.57%, and when the strain rate decreased from 10 to 0.1 s^−1^, the *R*_AV_ value rose from 4.28 to 7.69%, and the error gradually increased, so it was necessary to correct it. The error effect of true strain was even greater. When the deformation was small (less than 0.2), the *R*_AV_ value did not exceed 2.32%, and the influence was small. When the true strain exceeded 0.5, the error was as large as 11.0%. If the error was not corrected, it would not cause too much error, which would significantly affect the accuracy of industrial process design by numerical simulation.

**Table 2:** Average relative error before and after flow curve correction (*R*_av_, %).

Deformation condition	Deformation temperature/°C					Strain rate/s^−1^				True strain		
	800	900	1000	1100	1200	0.01	0.1	1	10	0–0.2	0.2–0.5	0.5–0.931
*R*_av_	4.54	5.35	5.59	6.61	7.57	7.69	7.10	4.64	4.28	2.32	6.45	11.0

## Comparison of simulation and test results

### Comparison of bulge sizes

In the process of thermal deformation compression, the formation of the bulge is related not only to the friction of the end surface but also to the internal temperature gradient of the sample. Therefore, the settings of the friction coefficient and heat conduction coefficient were crucial in the finite element simulation. However, whether the setting of the correlation coefficient was appropriate could be evaluated according to the comparison between the experimental and simulated results of the sample size after deformation. Table 3 shows the size of the deformed sample in the experimental and simulated results for strips with different deformation. The initial height of the specimen in the test was 12 mm, and the diameter was 8 mm. Due to the operation of the compression test and other reasons, the amount of compression in the test was not consistent. Therefore, in this study, only the effect of compression on the bulge size was compared. That is, this ensured that the amounts of compression were the same in the simulation and the test, and then the bulge sizes were compared. In Table 3, *H* is the height of the specimen after compression, and *D*_1_ and *D*_2_ are the diameters of the bulges in the test and simulation, respectively. A comparison showed that *D*_1_ and *D*_2_ were similar within the average relative error of only 1.06%, which indicated that the friction coefficient and heat conduction coefficient were set properly in the simulation. It also showed that the area of the bulge belly obtained by point tracking during the simulation was reliable.

**Table 3:** The size of the specimen after deformation in the experimental and simulation results for different deformation strips.

*T*/°C	0.01 s^−1^			0.1 s^−1^			1 s^−1^			10 s^−1^		
	*H*/mm	*D*_1_/mm	*D*_2_/mm	*H*/mm	*D*_1_/mm	*D*_2_/mm	*H*/mm	*D*_1_/mm	*D*_2_/mm	*H*/mm	*D*_1_/mm	*D*_2_/mm
800	6.0	12.05	11.99	6.0	12.54	11.95	5.6	12.48	12.11	5.5	12.55	12.18
900	6.0	12.12	12.21	6.0	12.31	12.11	5.9	12.06	11.83	5.5	12.32	12.16
1000	5.6	12.52	12.63	5.7	12.26	12.42	5.5	12.25	12.28	5.5	12.53	12.14
1100	5.5	12.67	12.78	5.6	12.15	12.64	5.5	12.24	12.3	5.5	12.47	12.13
1200	5.5	13.02	12.92	5.5	12.57	12.86	5.5	12.67	12.35	5.5	12.35	12.15

### Comparison of metal flow lines

**Figure 8:**
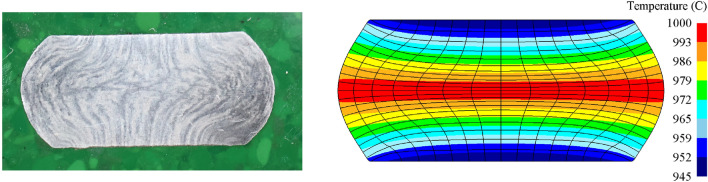
Metal streamlines after compression at 1000 °C and 0.1 s^−1^. (**a**) Test; (**b**) simulation.

During the process of hot deformation of metal materials, the grains are extruded and elongated along the direction of plastic forming under the action of external forces to form fibers. After proper corrosion treatment, streamlined streaks appear on the macroscopic level, namely, metal streamlines^18^. Figure 8 shows the distribution of compressed metal streamlines at 1000 °C and 0.1 s^−1^ under deformation conditions. Figure 8a shows the metal streamlines obtained by corrosion after the test, and Fig. 8b shows the distribution of metal streamlines obtained by simulation. The distribution of metal streamlines in the simulation results was basically consistent with that in the test. The metal flow lines of the upper and lower end faces of the sample were vertical due to the large friction between the specimen end face and the indenter, which made it difficult for horizontal deformation to occur at the specimen end face. On the whole, the grains closer to the center section of the sample were extruded and stretched more obviously along the normal direction of the forging surface, and the greater the curve of the metal flow line at the center section was from the center, the greater the bending degree. This showed that the displacement in the horizontal direction was greater at locations farther from the center.

In addition, Fig. 8b shows that after the sample was compressed, the internal temperature distribution was not uniform. This was because there was heat transfer between the end face and the indenter, which caused the temperature of the end face of the specimen to drop. In the thermal compression test, the temperature at the central cross section of the specimen should be kept constant by means of resistance heating. At the end of the test, the temperature was higher at the central cross section than at the two end faces, and the temperature gradient deepened the nonuniformity of the deformation of the specimen during the compression process.

### Comparison of load‒displacement curves

**Figure 9:**
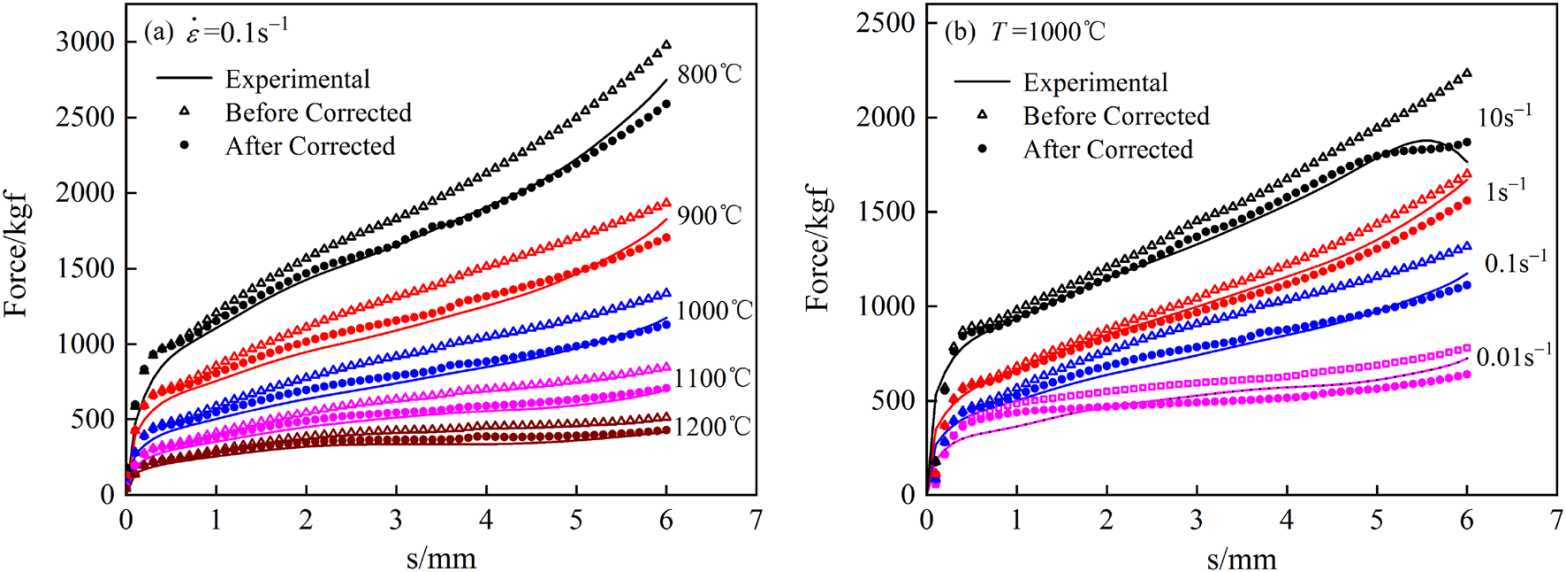
Comparison of load displacement diagrams for the test and finite element analysis. (**a**) 0.1 s^−1^; (**b**) 1000 °C.

The revised flow curve was substituted into the finite element method for the re-simulation calculation, and then the load‒displacement data from the two simulation results, before and after correction, were derived and compared with the experimental load, as shown in Fig. 9. Figure 9 shows that the load obtained by direct simulation before correction was larger than that in the test, and the load value obtained by simulation again after correction was similar to that in the test. This indicated that there was a large error in the flow curve before correction, and the flow curve after correction was very similar to the real flow curve of the material.

## Conclusions

Accurate stress‒strain data play an important role in hot compression tests. In this paper, the flow curve of 34CrNi3MoV steel was obtained. In view of the nonuniformity of specimen deformation during compression, the flow curve was modified by the finite element method. The main conclusions can be summarized as follows:

1. Bulging is obvious in the deformation of the specimen during compression, and bulging has a significant effect on the stress during large deformation, which cannot be ignored.
2. The flow curves modified by the finite element method are all lower than the original curves. At the beginning of deformation, the difference between the two is small, but with the increase in deformation, the uncorrected error will gradually increase.
3. The bulge size and metal flow line in the finite element simulation results are basically consistent with the test results, and the error of the size of the bulge is only 1.06%, which indicates that the area value of the bulge obtained by using point tracking in the simulation process is reliable.
4. The modified flow curve is substituted into the finite element method for the resimulation calculation, and the load value obtained is basically close to the load value in the test, indicating that the modified flow curve is very close to the real flow force curve of the material, which also shows that the method is feasible. The research in this paper has laid a good foundation for more accurate analyses, such as constitutive equation construction, recrystallization experiments and finite element simulations.

## Data Availability

The data that support the findings of this study are available from the corresponding author upon reasonable request.
